# The effects of downhill and uphill exercise training on osteogenesis-related factors in ovariectomy-induced bone loss

**DOI:** 10.20463/jenb.2017.0010

**Published:** 2017-09-30

**Authors:** Yun-Seok Kang, Chun-Ho Kim, Jeong-Seok Kim

**Affiliations:** 1. Department of Sports Science, Chonbuk National University, Jeonju Republic of Korea; 2. Department of Physical Education, Chonbuk National University, Jeonju Republic of Korea

**Keywords:** Ovariectomized rat, Bone formation, Osteogenesis, Bone mineral density, Trabecular bone

## Abstract

**[Purpose]:**

Recent evidence suggests that regular exercise training plays a decisive role in maintaining homeostasis and promoting muscle and skeletal formation. However, the effect of downhill exercise training on osteogenesis-related factors is not well understood.

**[Methods]:**

Thus, we investigated the effect of uphill and downhill training on ovariectomy (OVX)-induced bone loss. After ovary removal, the exercise method performed included uphill (16 m/min, +15°) and downhill training (16 m/min, –15°) for 60 min/day and 5 days/week, respectively, for 8 weeks.

**[Results]:**

Our results showed that both uphill and downhill training significantly decreased the body weight, total cholesterol, and creatine kinase (CK) levels in the context of OVX-induced bone loss. On the contrary, levels of an osteogenesis indicator, osteocalcin and alkaline phosphatase were elevated. Consequently, the uphill and downhill training reduced OVX- induced bone loss in the distal femoral metaphysis. Likewise, the bone microstructure in OVX-induced bone loss was enhanced upon training. In particular, the inhibition of RANKL-induced osteoclast formation and osteoclast-specific gene expression improved upon downhill training compared to uphill training.

**[Conclusion]:**

These results suggest that the uphill and downhill exercise types appeared to positively affect the expression of osteogenesis-related factors along with bone density and microstructure. Particularly, the downhill training has more beneficial effects on the maintenance of homeostasis during bone formation.

## INTRODUCTION

Osteoporosis is a metabolic bone disease which results in marked decrease in the bone mineral density (BMD) and bone strength in patients due to reduction of bone components, as compared to age-and gender-matched healthy persons^1^. Moreover, it can increase the frequency of fractures, thereby contributing to associated problems including difficulties with treatment and high cost upon occurrence of a fracture^2^.

Bones undergo a repeated cycle of bone formation by osteoblasts followed by bone resorption by osteoclasts, and this mechanism, in an active manner, treats micro-injuries to bones and replaces old bone tissues with new ones^3^. Both males and females see maximum increase in BMD in their mid-30’s, with females experiencing a decrease of about 1.2% per year and a rapid decrease of 8% per year after menopause^4^. This rapid decrease is attributed to the changes in secretion of female hormones due to ovarian hypofunction, and among which the decreased secretion of especially estrogen has been reported as the primary cause of osteoporosis in post-menopausal women^5^. Estrogen is an important factor that binds to estrogen receptors on osteoblasts to directly affect bone metabolism, while it is also known to facilitate bone formation by playing a role in calcium resorption in various organs^6^. Moreover, low estrogen level hampers the activation of lipoprotein lipase (LPL), and thereby its induction, which reduces the lipolytic function to cause fat accumulation^7^. Furthermore, increased body fat leads to increased production of pro-inflammatory cytokines, such as interleukin-6 (IL-6) and tumor necrosis factor-alpha (TNF-α) that facilitates bone resorption through activation of osteoclasts^8^. Differentiation of precursor cells to osteoclasts requires two types of cytokines; receptor-activated nuclear factor κB ligand (RANKL) and macrophage colony-stimulating factor (M-CSF)^9^. In particular, RANKL is an important cytokine required for osteoclast differentiation, while M-CSF is essential for the survival and proliferation of osteoclast precursors^10^. Inhibitory effects on these osteoclasts are associated down regulation of major signaling pathways, such as NF-κB, JNK, p38, Akt, c-Fos, and NFATc1^9^, ^11^. Therefore, decreased estrogen secretion due to menopause causes a notable increase in bone loss on the surface of cortical and trabecular bones through imbalance between bone resorption and formation, which ultimately leads to osteoporosis^12, 13.^

Once osteoporosis occurs, there are no safe and effective methods to recover normal bone tissues, and thus, prevention should be given priority over treatment. Moreover, since bone loss is especially high in the first 3–5 years after menopause, this period is critical to adopt the necessary precautionary measures^14^. Current treatment modalities for improving bone health include estrogen replacement therapy and use of bisphosphonate class of drugs for inhibiting or slowing the progression of bone loss. However, because these methods have been reported to have low efficacy and possible adverse effects from long-term use, including breast cancer and hypertension^15^, safer and more effective treatment modalities are required.

Physical activities and exercise are known to promote bone metabolism by increasing the load being exerted on the bones, and have also been reported to be effective in maintaining the peak BMD in young people and inhibiting BMD reduction associated with aging^16^. Among them, walking and running are weight-bearing exercises that are effective in maintaining and improving muscle functions and BMD^17^. Such weight-bearing exercises promote bone metabolism because of the exertion of a physical impact on the bones and increase BMD by inducing formation of muscles that are responsible for the support functions of bones, while also reducing the risk of injuries, such as fractures^18^. A study by Sun et al. (2015) subjected menopause-induced rats to eight weeks of high-intensity exercise and reported positive effects on BMD and bone microstructure based on the findings of increased femoral BMD level, along with a significant increase in the percent bone volume (BV/TV), trabecular thickness (Tb.Th), and trabecular number (Tb.N)^19^. It was further reported that increase in the expression of bone resorption markers induced by menopause in blood decreased significantly after exercise therapy, indicating aerobic exercise inhibited the bone resorption process. However, Miyatake et al. (2016) reported that 6 weeks of moderate-intensity aerobic exercise resulted in a significant increase in the femoral and tibial microstructures in the normal group, whereas no significant differences were found in the menopause-induced group^20^. Therefore, it is necessary to clearly analyze how the differences in the intensity and type of exercise affect the factors related to post-menopausal bone metabolism when estrogen secretion is decreased, and infer an appropriate exercise program from the findings.

Accordingly, the present study prepared an ovariectomized, menopause-induced model with the objective of investigating the effects of downhill and uphill types of aerobic exercises on changes in the concentrations of blood estrogen and bone metabolism markers, BMD, and bone microstructure, along with differentiation of osteoclasts derived from femoral bone marrow.

## METHODS

### Experimental animals

The experimental animals used in the present study were female Sprague-Dawley rats (n = 32) with the same birth period (8 weeks old), which were procured from Damool Science. The experimental animals were reared in experimental animal center after obtaining an approval from the Institutional Animal Care and Use Committee of “J” University (CBNU 2016-0029). The temperature and humidity in the animal room were set to 23–25°C and 70–80%, respectively, while light-darkness regime was adjusted to a 12-hr cycle. The experimental animals were supplied with enough water and solid feed (22.5% protein, 3.5% fat, 7.0% low-fiber, 9.0% ash, 0.7% Ca, and 0.5% P; Damool Science, Daejeon, South Korea) throughout the entire experimental period.

### Experimental methods

#### Ovariectomy

After 1-week acclimation period, the experimental animals were randomly assigned into a non-ovariectomized sham control group (Sham-Con; n = 8), ovariectomized control group (OVX-Con; n = 8), and two aerobic exercise groups of ovariectomized uphill exercise group (OVX-E1; n = 8) and ovariectomized downhill exercise group (OVX-E2; n = 8). All experimental animals were anesthetized by an intraperitoneal injection of a general anesthetic agent (1mL/kg body weight), which was a mixture of Zoletil (Virbac S.A, Carros cedex, France), Rompun (Bayer Korea, Seoul, Korea), and saline solution in a 2:1:2 ratio. In the ovariectomized groups, a skin incision was made on the center of the back where the hair had been removed; skin and muscles on both sides of the back were excised; both ovaries were excised; and the site was sutured. In the non-ovariectomized group, a sham operation performed, in which the hair removal and skin incision on the center of the back were performed in the same manner, and the site was sutured after only removing the muscles on both sides. Povidone iodine solution was applied on the surgical site to prevent infection. Subsequently, the experiment was conducted after two weeks of recovery period.

#### Exercise methods

The exercise groups underwent 1 week of acclimation training on a rodent treadmill (Omnipacer model LC-4, Omni tech columbus) for 10–15 min/day, with the settings of 5–10 m/min and 0˚ incline. After this acclimation period, the exercise groups performed 8 weeks of incremental treadmill exercise with maximum exercise intensity of 16m/min at an incline of 15° (concentric exercise; uphill) and 16m/min at a decline of -15° (eccentric exercise; downhill), which was performed 60 min per day and 5 days per week^21, 22, 23, 24.^

#### Tissue harvesting

Upon completion of the 8-week exercise program, the experimental animals were anesthetized peritoneal and blood was collected from the abdominal aorta. Blood samples were centrifuged for 30 min (3,000 rpm, 4°C) to separate the serum, which was stored at –80°C until further analysis. For analysis of BMD and bone microstructure, the right thigh was stored in a tube containing 4% formalin. For osteoclast culturing, left femur was separated and bone marrow cells (BMCs) were obtained by washing the bone pulp using a 1 mL syringe.

### Analysis methods

#### Blood biochemistry analysis

Serum estradiol, osteocalcin, and alkaline phosphatase levels were measured using an ELISA Kit (MyBio-Source Inc., San Diego, CA, USA). After dispensing 25 μL of standard solution and serum from each sample to the antibody-coated wells, 100 μL of working reagent conjugate buffer was added and mixed for 20 sec, followed by culturing for 2 hr at 37°C. After removing the contents of each well, a multichannel pipette was used to repeatedly wash each well 3 times with 300 μL of wash buffer. In the final step, the remaining wash buffer and bubbles were removed from the well, after which, 100 μL of TMB reagent was dispensed to each well and mixed thoroughly for 10 sec, followed by culturing for 30 min at 37°C. After dispensing 50 μL of stop solution and mixing for 30 sec, an ELISA reader (Model 550 Microplate reader, Bio-Rad Inc., Hercules, California, USA) was used to measure the absorbance at 450 nm within 10 min and the values were substituted into the standard curve for calculation. Serum creatine kinase (CK) activity was analyzed via fluorescence intensity using REP CK Kit (Helena, U.S.A), while serum total cholesterol (TC) level was measured using T-CHO kit (3I2020, Asanpharm, Hwaseong, Korea). Cholesterol esterase breaks down ester type cholesterol into free cholesterol and fatty acid. H2O2 produced from oxidation by cholesterol oxidase produces red quinine pigment by oxidative condensation of 4-aminoantipyrine and phenol by peroxidase. This pigment was used to measure the absorbance at 500 nm with an ELISA reader to derive the blood TC level. To 20 μL of serum and standard solution, 3 mL of enzyme solution was admixed and incubated for 5 min at 37°C, after which, the absorbance at 500 nm within 60 min against a blank control was measured using an ELISA reader and the calculations were made by the equation given below:

TC Level (mg/dl) = (specimen absorbance / standard absorbance) × 300 (mg/dl)

#### BMD and microstructure analysis

For BMD and bone microstructure of the femur, micrographic images were acquired by using micro-computed tomography (micro-CT, Skyscan 1076, Belgium) to irradiate an X-ray (voltage of 100 kA and current of 100 μA) that penetrated the specimen through a 0.5 mm aluminum filter. Acquired images were reconstructed to gray scale level using Nercon Ver 1.3 (Skyscan), and the reconstructed 2D images were reproduced as 3D models using CTAn and CTVox (Skyscan) software. CTAn software analyzed the images by setting the region of interest (ROI) spanning from 0.5 mm to 4 mm away from the growth plate of the measured femur. Measurements of BMD, along with Tb.Th, Tb.N, Tb.Sp, and BV/TV were taken within the volume of interest (VOI), which was the morphological microstructure of bone. CTVox software was used for the imaging of 2D cross-sections and 3D structures of bony trabecula within the femur.

#### Osteoclast differentiation

BMCs obtained from rat femur were cultured for 12 hr in an α-MEM medium containing 10% FBS and antibiotics, and on the following day, the experiment was conducted using the cells that did not adhere to the bottom of 10 cm plate considered as bone marrow-derived macrophages (BMMs)^25^. Collected BMMs were dispensed into a 24-well plate (2 × 105 cells/well) together with M-CSF (30 ng/mL) and cultured for 3 days. After 3 days, the BMMs were treated with M-CSF (30 ng/mL) and RANKL (50 ng/mL). At 3 days after culturing, the differentiated cells were stained with tartrate-resistant acid phosphatase (TRAP) staining kit (387A-1KT; Sigma Aldrich, USA) and red-colored differentiated osteoclasts were quantitatively analyzed. Moreover, the number of osteoclasts and their area were measured by a method proposed in a previous study, which involved observation using Axiovert 40 CFL microscope (Carl Zeiss, Oberkochen, Germany) and measurement using iSolution DT 36 software (Carl Zeiss)^26^.

#### Quantitative real-time PCR analysis

TRIzol (Invitrogen, USA) was used for extraction of total RNA from cells, while QuantiTect Reverse Transcription Kit (Takara, Japan) was used for reverse transcription of cDNA from 2 μg of RNA. Quantitative real-time PCR was performed in ABI 7500 using Power SYBR Green PCR Master Mix (Takara, Dalian). The relative amount of mRNA normalized by β-actin was calculated using the delta-delta method. PCR primer sequences were as follows:

c-Fos: 5′-CCAGTCAAGAGCATCAGCAA-3′ (forward) and 5′-AAGTAGTGCAGCCCGGAGTA-3′ (reverse); NFATc-1: 5′-CCGTTGCTTCCAGAAAATAACA-3′ (forward) and 5′-TGTGGGATGTGAACTCGGAA-3′ (reverse); β-actin: 5′-ACCCAGAAGACTGTGGATGG-3′ (forward) and 5′-CACATTGGGGGTAGGAACAC-3′ (reverse).

#### Data processing methods

Means and standard deviations were derived from the measurement data using SPSS Win 23.0 statistics program, and a one-way ANOVA was used for testing the mean differences between groups. The hypothesis acceptance level was set to p<0.05, and if statistically significant differences were found in the analysis, post-hoc test was performed via Student Newman-Keul method.

## RESULTS

### Body weight and expression of blood-related markers in ovariectomized rats according to type of exercise

**Figure 1.**
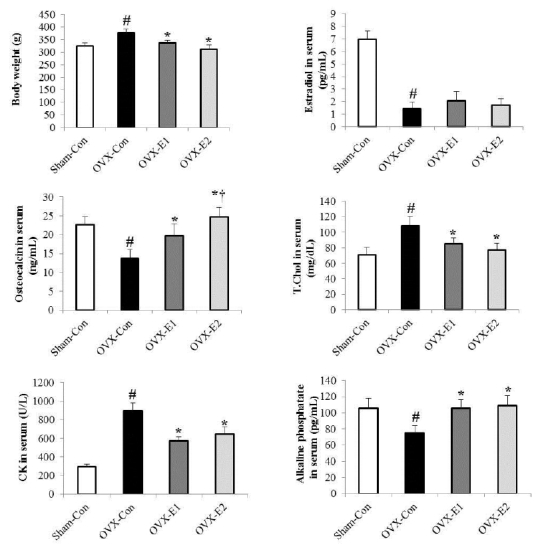
The body weight and serum estradiol, osteoclacin, total cholesterol, creatine kinase (CK), and alkaline phosphatase(ALP) determines in the uphill and downhill exercise training. Values represents the mean±SE; The symbol ＊indicates statistically significant differences between OVX-Con and OVX-E1 groups p<.05. The symbol † indicates statistically significant difference between OVX-E1 and OVX-E2 groups p<.05. The symbol # indicates statistically significant difference between Sham-Con and OVX-Con groups p<.05.

Body weight and expression of blood-related markers in the experimental animals were as shown in Fig. 1. Body weight and blood concentrations of TC, osteocalcin, alkaline phosphatase, and CK in the experimental animals were significantly different in OVX-Con group compared with Sham-Con, OVX-E1, and OVX-E2 groups (Fig 1. p<0.05). However, for the blood concentration of estradiol, OVX-Con group showed a significant difference only against Sham-Con group (Fig 1. p<0.05). Blood concentration of bone formation markers, osteocalcin and alkaline phosphatase, were significantly increased in OVX-E1 and OVX-E2 groups, as compared to OVX-Con group (Fig 1. p<0.05), and in particular, concentration of osteocalcin showed significant increase as a result of the exercise type in OVX-E2 than in OVX-E1 group (Fig 1. p<0.05).

### Changes in BMD and bone microstructure in ovariectomized rats according to type of exercise

**Figure 2.**
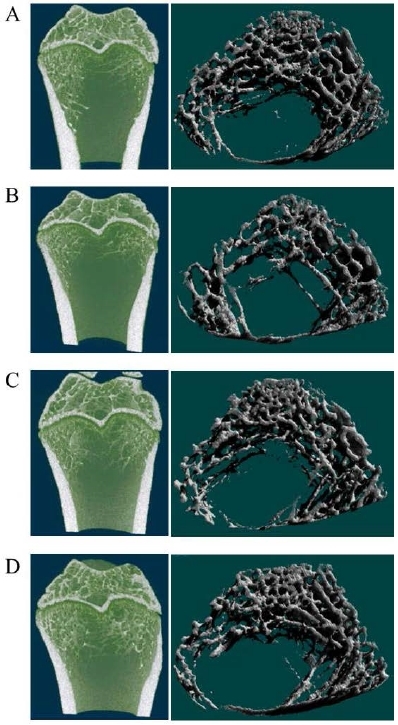
The uphill and downhill exercise training reduces OVX-induced bone loss in the distal femoral metaphysis. A. Sham-Con. B. OVX-Con. C. OVX-E1. D. OVX-E2. Representative micro-CT pictures are shown.

**Figure 3.**
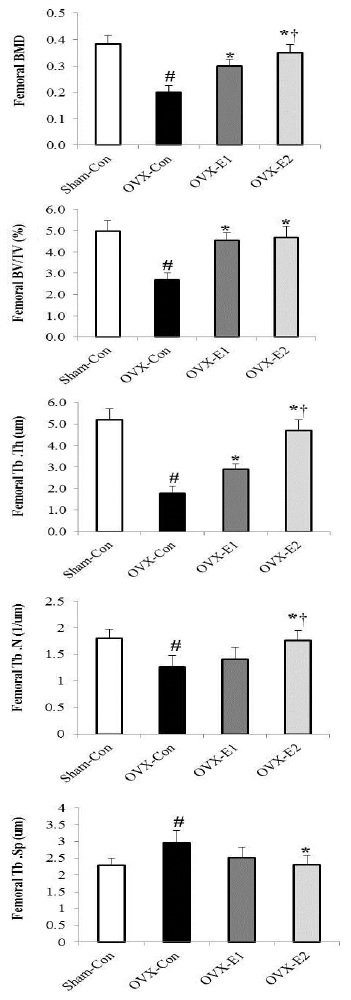
The uphill and downhill exercise training enhances bone microstructure in OVX-induced bone loss. Femoral bone mineral density (BMD), bone volume/tissue volume (BV/TV), trabecular thickness (Tb.Th.), trabecular number (Tb.N.), and trabecular separation (Tb.Sp.) were quantitatively analyzed using the micro-CT system. Values represents the mean±SE;＊indicates statistically significant differences between OVX-Con and OVX-E1 groups p<.05. The symbol † indicates statistically significant difference between OVX-E1 and OVX-E2 groups p<.05. The symbol # indicates statistically significant difference between Sham-Con and OVX-Con groups p<.05.

Fig. 2 shows the morphological changes in the femur according to ovariectomy status and exercise type, while Fig. 3 shows changes in BMD and bone microstructure. With respect to morphological changes in the femur, both OVX-E1 and OVX-E2 groups showed less bone loss than OVX-Con group, and in particular, bone loss appeared lower in OVX-E2 group than in OVX-E1 group (Fig 2). Moreover, BMD, BV/TV, Tb.Th, and Tb.N had significantly lower values in OVX-Con group than in Sham-Con, OVX-E1, and OVX-E2 groups (Fig 3. p<0.05), whereas Tb.Sp was significantly higher in OVX-Con group compared to others (Fig 3. p<0.05). Especially BMD, Tb.Th, and Tb.N showed significant differences between OVX-E1 and OVX-E2 groups (Fig 3. p<0.05).

### Osteoclast formation and expression of related factors in ovariectomized rats according to type of exercise

**Figure 4.**
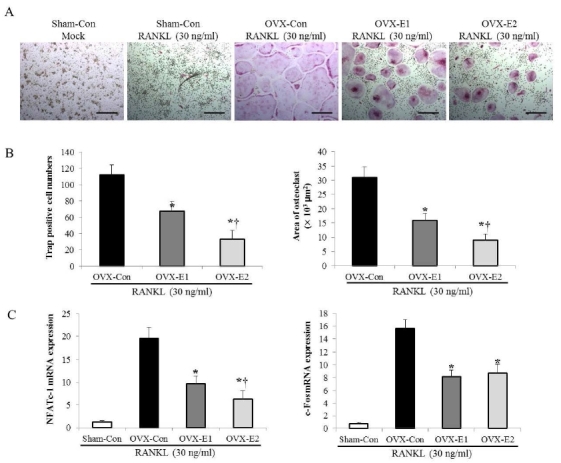
The uphill and downhill exercise training inhibits RANKL-induced osteoclast formation and osteoclast-specific gene expression. A. BMMs of the uphill (OVX-E1) and downhill exercise (OVX-E2) groups were treated with the M-CSF (30 ng/mL) and RANKL (50 ng/mL) and were stained for TRAP activity. Scale bar = 200 μm in representative images). B. The osteoclasts number and the area were determined. The data represents three repeated experiments and are shown as the mean ± SD. C. Total RNA was extracted, and the mRNA level of NFATc-1 and c-FOS were measured by real-time PCR. β-actin was used as an internal control. Values represents the mean± SD; ＊indicates statistically significant differences between OVX-Con and OVX-E1 groups p<.05. The symbol † indicates statistically significant difference between OVX-E1 and OVX-E2 groups p<.05.

Fig. 4 shows the changes in osteoclast formation and expression of related factors in ovariectomized rats according to ovariectomy status and exercise type. Differentiation of mature osteoclasts based on TRAP-staining of BMMs showed different inhibition patterns according to the type of exercise (Fig 4. A and B; p<0.05). In particular, differentiation form, numbers, and area of osteoclasts were much lower in OVX-E2 group than in OVX-E1 group (Fig 4. A and B; p<0.05). Moreover, NFATc1and c-Fos, known osteoclast differentiation-specific transcription factors, showed different inhibition patterns based on the type of exercise (Fig 4. C; p<0.05). In particular, NFATc1 showed significantly lower osteoclast differentiation in OVX-E2 (downhill) group than in OVX-E1 (uphill) group (Fig 4. C; p<0.05).

## DISCUSSION

In the present study, ovariectomy was performed to induce physiological characteristics similar to menopause with the objective of investigating the effects of aerobic exercise type (uphill and downhill) on the changes in bone metabolism and bone microstructure of menopausal models. Menopause is a condition in which estrogen secretion is decreased due to ovarian hypofunction caused by aging, and it diminishes lipid and bone metabolism, while increasing the prevalence of obesity and osteoporosis^27^. Such decreased estrogen secretion due to ovarian hypofunction restricts the activation of low-density lipoprotein cholesterol receptors and increases the blood lipid and cholesterol levels, which can cause excessive fat accumulation^28^. Moreover, this leads to increase in the expression of inflammatory cytokines, such as IL-1, IL-6, and TNF-α, to induce differentiation from mesenchymal stem cells to osteoclasts, which can facilitate decrease in BMD^29^.

The results in the present study showed that OVXCon group had significantly different body weight and higher blood TC and CK levels than the Sham-Con group. On the other hand, OVX-E1 and OVX-E2 groups, which performed 8 weeks of exercise, showed reduced levels that were similar to Sham-Con group. These results indicated that both types of aerobic exercises (uphill and downhill) were effective for cases of increased body weight and blood lipid level as well as decreased CK level caused by ovariectomy. However, blood estradiol concentration did not show an increased pattern upon exercise. The results from the study by Kim (2012) that subjected ovariectomized rats to 8 weeks of moderate-intensity aerobic exercise^30^ and study by Suk et al. (2009) that applied 16 weeks of resistance exercise on menopause group, showed increase in body weight and blood lipid levels in the experimental group as compared to the control group. However, no significant differences in blood estradiol concentration were observed in these studies, which were similar to the results in the present study^31^. In the study by Li et al. (2014), blood estradiol concentration increased significantly in ovariectomized rats after treatment using voluntary wheel running exercise, and such changes in concentration was attributed to the appropriate exercise intensity^32^. These results supported the findings by Kim (2012) and Scott et al. (2014) on moderate-intensity aerobic exercise using a treadmill^30, 33^ and Stringhetta-Garcia et al. (2016) on resistance exercise showed that the serum estradiol levels were decreased or similar compared to the levels of control group (ovariectomized) ^34^. Therefore, it is believed that additional studies in accordance with the exercise intensity are needed to accurately identify factors that impact lipid and bone metabolism and changes in the blood estradiol concentration based on exercise after ovariectomy.

Diagnosis, evaluation, and treatment response for osteoporosis can be assessed by analysis of bone metabolism related indicators in blood, which consist of bone matrix components released during bone resorption and formation, and enzymes secreted by osteoclasts and osteoblasts that can be measured in blood or urine^35^. Osteocalcin, an indicator of bone formation, is a vitamin K-dependent protein that is secreted mostly by osteoblasts, and because approximately 30% of osteocalcin produced in osteoblasts are released into the blood, it allows the determination of osteoblast activities^36, 37.^ Moreover, alkaline phosphatase is a target enzyme of the parathyroid hormone and is a glycoprotein secreted by osteoblasts that is the most commonly used bone formation indicator in clinical settings^38^. In a study by Tartibian et al. (2011), blood osteocalcin concentration increased in menopausal women after 12 weeks of moderate-intensity aerobic exercise^39^, while in a study by Iwamoto et al. (2004), blood osteocalcin concentration increased significantly in ovariectomized rats after 7 and 11 weeks of high-intensity aerobic exercise^40^. In the present study, bone formation indicators, osteocalcin and alkaline phosphatase, showed significant reduction in OVX-Con group, as compared to Sham-Con group, while showing significant increase in OVX-E1 and OVX-E2 groups. These results indicated that decreased estrogen secretion due to ovariectomy restricted the activities of osteoblasts, while 8 weeks of aerobic exercise facilitated bone formation by osteoblasts. In addition, increase in the bone formation markers without significant changes in blood estrogen levels based on exercise therapy demonstrate the positive effects of continued aerobic exercise on bone metabolism in the context of the condition of decreased estrogen secretion due to ovariectomy. In particular, increased osteocalcin concentration from downhill aerobic exercise, an eccentric exercise, is a noteworthy result, which is believed to be effective in reducing bone loss due to aging of the body.

Long bones in the body are composed of cortical bones that make up the hard outer area and cancellous bones that constitute the soft center, and damages to bone tissues caused by osteoporosis usually occur in the cancellous bones^41^. In menopausal women, increased bone resorption causes not only decreased BMD in cancellous bones, but also wider trabecular separation from large decrease in the trabecular number and size within cancellous bones, which results in easy occurrence of fractures even from a small impact and requiring longer recovery period^42^. In precedent studies, Tartibian et al. (2011) reported that aerobic exercise was effective in improving BMD in menopausal women^39^, while Sun et al. (2015) reported that 8 weeks of high-intensity aerobic exercise showed effects of decreased BMD and increased BV/TV, Tb.Th, and Tb.N, which are bone microstructure indicators, in ovariectomized rats^19^. The present study showed that femoral BMD, BV/TV, Tb.Th, and Tb.N decreased significantly in ovariectomized OVX-Con group than in Sham-Con group, while Tb.Sp level was increased due to decreased trabecular size and number by the inhibition of estrogen secretion. On the other hand, both uphill and downhill aerobic exercise for 8 weeks showed a highly positive effect on the changes in BMD and bone microstructure. In particular, BMD and bone microstructure similar to Sham-Con group appeared more frequently in eccentric downhill exercise group than in concentric uphill exercise group. Eccentric exercise at appropriate intensity level has been suggested as being helpful in achieving positive physiological effects in aged rats, such as maintaining homeostasis and protecting skeletal muscles and bones without various complications associated with high-intensity exercise^22, 23.^ Therefore, in order to improve the BMD and bone microstructure weakness due to ovariectomy and menopause, high intensity eccentric exercise that exerts sufficient stimulation to the bones would be effective. However, since concentric exercise can also have an effect on improving the bone health, maintaining an exercise regimen that is performed regularly and at appropriate intensity level, with consideration for one’s own health status, should play an important role in bone formation and maintenance.

Bone formation and maintenance are known to be regulated by osteosynthesis by osteoblasts and resorption by osteoclasts^43^. Inhibitory effects of osteoclasts affect bone formation and synthesis by inducing the inactivation of regulators of major signaling pathways for osteoclast formation, such as c-Fos, receptor activator of nuclear factor (NF-κB) ligand (RANKL), and its receptor RANK^9^. Among these, RANKL is known to play a crucial role in bone resorption by inducing activation, formation, and differentiation of osteoclasts^44^. Previous study on bone formation (trabecular) through exercise also suggested that regular swimming exercise can have a positive effect on BMD and bone mass in cases of bone weakening and loss due to ovariectomy^45^. In the present study, BMCs obtained from the femur were treated with RANKL, which promotes osteoclast differentiation, for the measurement of osteoclast differentiation and expression of related factors based on 8 weeks of aerobic exercise. The results were consistent with previous studies, showing significant decrease in the osteoclast area and TRAP, which has increased expression in mature osteoclasts in the groups that underwent exercise^46^. Moreover, mRNA levels of c-Fos and NFATc1, known upstream factors of TRAP that promote differentiation from preosteoclasts to osteoclasts^47^, were significantly reduced in the groups that underwent exercise. In particular, TRAP activity, osteoclast area, and NFATc1 mRNA levels significantly decreased with downhill exercise compared to uphill exercise. Stringhetta-Garcia et al. (2016) reported that exercise causes not only effective changes in BMD and bone microstructure, but also significant decrease in RANKL and TRAP, differentiation factors of osteoclasts in menopause-induced rats that underwent 16 weeks of resistance exercise^34^. However, Wang et al. (2013) reported that aerobic exercise using a treadmill (18 m/min, 0°, 45 min, 11 weeks) resulted in significant increase in mRNA expression of OPG, an inhibitory factor for osteoclast differentiation in the femur, whereas osteoclast differentiation promoting factors, RANKL and RUNX2, decreased as compared to the control group, but without significant differences^48^. Such results from precedent studies suggest that inhibition of osteoclast differentiation that can affect BMD and bone microstructure may change according to the intensity and type of exercise. In the present study, both uphill and downhill exercise groups were effective in inhibiting osteoclast differentiation, and in particular, downhill exercise involving eccentric contraction was more effective than uphill exercise involving concentric contraction in decreasing mature osteoclast activities. Therefore, it is believed that various future studies are needed to find effective exercise methods for improving bone health that may have been diminished by menopause.

In conclusion, the present study generated a menopause-like model via ovariectomy to observe the effects of aerobic exercise based on type of exercise from the aspects of bone metabolism markers, BMD, bone microstructure, and osteoclast differentiation. The findings in the present study suggested that regular aerobic exercise can have a positive effect on bone health in the context of restricted estrogen secretion due to menopause.
